# Sibling Relationships in Families of Autistic and Typical Children: Similarities and Differences in the Perspectives of Siblings and Mothers

**DOI:** 10.1007/s10803-023-06222-0

**Published:** 2024-01-20

**Authors:** Yonat Rum, Ditza A. Zachor, Yael Armony, Ella Daniel, Esther Dromi

**Affiliations:** 1 The Hebrew University of Jerusalem, Jerusalem, Israel; 2 University of Cambridge, Cambridge, UK; 3 Shamir (Assaf Harofeh) Medical Center, Zerifin, Israel; 4 Tel Aviv University, Tel Aviv, Israel

**Keywords:** Autism, Siblings, Sibling relationship, Multiple reporters

## Abstract

This study investigates mothers' and siblings' perspectives regarding similarities and differences in siblingships with and without autism. Twenty-nine typical children (*M*_age_ = 8.78 years, *SD* = 2.05) whose younger siblings have a diagnosis of autism and their mothers constituted the ‘autism group.’ Forty-six typical children (*M*_age_ = 9.12 years, *SD* = 2.06) with younger typical siblings and their mothers constituted the ‘typical group’. Children and mothers completed the Sibling Relationship Questionnaires and were interviewed. Children's reports illustrated similarities in sibling relationships across groups, with no between-group differences in overall warmth, conflict, rivalry, or relative power in the sibling relationship. Mothers of autistic children reported their children to have less warmth and closeness (*F*_(1,72)_ = 5.63, *p* = .02, η^2^ = .073) and less conflict (*F*_(1,72)_ = 6.66, *p* = .012, *η*^2^ = .085) with their siblings than mothers of typical children. More disagreement was found between mothers and children in the autism group than in the typical group. Mothers and children reported less intimacy and less quarreling between the siblings in the autism group than in the typical group. The qualitative analysis suggests that siblings focused on the present experience, while mothers also referred to processes in the sibling relationship. Interviewees in the autism group referred to various specificities in the siblingships without explicitly using the term “autism”. The findings highlight a typical-like experience for siblings of autistic children. At the same time, mothers' perspectives emphasize differences and challenges in siblingships, reporting a pattern of decreased involvement. It is important to consider both perspectives in research and clinical work with families of autistic children.

**Supplementary Information:**

The online version contains supplementary material available at 10.1007/s10803-023-06222-0.

## Introduction

Siblingship, a relationship with a sibling, is a meaningful relationship influencing development, psychosocial functioning, and well-being (Brody, 2004; Dunn, 2007; McHale et al., 2012; Noller, 2005). For autistic individuals, siblings might be a potential moderating (buffering) on the negative impact of social isolation: siblings begin as play partners in early childhood and can become a source of support throughout life (Gass et al., 2007; Stocker et al., 2020). Such roles might be particularly essential for autistic individuals who are likely to experience social challenges throughout life (Hendricks & Wehman, 2009; Ozonoff et al., 2010; Zwaigenbaum et al., 2005).

The social challenge that autistic individuals experience is traditionally referred to as an impairment (APA, 2022). However, accumulating evidence suggests that individuals on the autism spectrum may possess social abilities but often face specific challenges in this domain and that these abilities' manifestations also depend on their social partner’s characteristics and attitudes (e.g., Crompton et al., 2020b; Kimhi & Bauminger-Zviely, 2012; Morrison et al., 2019). As siblings share an environment and routine and form companionship, siblingships might be an optimal relationship for autistic individuals. The unique role of siblingship for autistic children was acknowledged in previous research (Ben-Itzchak et al., 2018; Knott et al., 2007; Rum et al., 2021).

*The Double Empathy Problem* theory (Milton et al., 2020) suggests that difficulties in relationships between autistic and non-autistic people are mutual and bidirectional. It is not only that autistic people struggle socially, but also, non-autistic people struggle to communicate and maintain relationships with autistic social partners. Considering siblingships within this framework places special importance on studying non-autistic siblings’ perspectives on the relationship. For typical siblings of autistic children, both negative and positive siblingship experiences were recorded (e.g., Ross & Cuskelly, 2006; Mascha & Boucher, 2006; see reviews: McHale et al., 2016 and Orsmond & Seltzer, 2007a, 2007b), but little research has focused on the relationships' qualities and the similarities and differences between these relationships and typical sibling relationships (Kaminsky & Dewey, 2001; Walton & Ingersoll, 2015).

### Similarities and Differences in Sibling Relationships in Families of Autistic and Typical Children

Throughout middle childhood, typical sibling relationships are characterized across families as generally positive, with high levels of warmth and closeness, alongside conflict (Buhrmester & Furman, 1990). As the difficulty in social communication is one of the core diagnostic features of autism (APA, 2022), one might hypothesize that autistic children and their siblings will have challenges building and maintaining their relationships.

In a meta-analysis focused on psychological outcomes in siblings of children on the autism spectrum, Shivers et al. (2019) found that the odds of individuals having “more negative” relationships with their autistic siblings were 1.5 to 3.0 times the odds of comparison groups. This effect was found in comparing siblings of autistic individuals not only to typical sibling pairs but also to siblings of individuals with other conditions. Thus, the researchers concluded that social communication deficits of the autistic sibling create challenges to the relationship. However, most studies in this meta-analysis that included measures of sibling relationships did not compare siblingships with and without autism. Some compared siblingships with autism only to sibling relationships in which one of the siblings has Down Syndrome (DS; Hodapp & Urbano, 2007; Orsmond & Seltzer, 2007a, 2007b; Pollard et al., 2013) or other disabilities (Pilowsky et al., 2004; Tomeny et al., 2012, 2017) and some included no comparison group (Granat et al., 2012; Hastings & Pet al.,as, 2014; Stampoltzis et al., 2014). Also, in the available literature, it is hard to find a reference to specific qualities of sibling relationships that could help us understand *what* is unique (or “more negative”) in siblingships of autistic children and what aspects of the relationship are shared with typical siblingship. Answering such questions requires: a. collecting data about the sibling relationship qualities and b. comparing siblingships in families with and without autism.

One such study was conducted by Kaminsky and Dewey in the year 2001. They compared self-reports of siblings (8–18 years old) of children on the autism spectrum to siblings of children with DS and pairs of typical siblings (30 participants in each group). Referring to the four factors of the Sibling Relationship Questionnaire (SRQ; Furman & Buhrmester, 1985): warmth and closeness, conflict, rivalry, and power (dominancy between the siblings), they found no group differences in power and rivalry and no difference between siblings of autistic children and siblings of typical children in closeness (siblings of children with DS reported more closeness than participants in the other two groups). Siblings of autistic children (as well as siblings of children with DS) reported less conflict than siblings of typical children. In the SRQ subscales, siblings of autistic children reported less intimacy in the siblingship and less nurturance by their siblings than siblings in the other groups. Additionally, siblings of autistic children (and siblings of children with DS) reported greater admiration of their sibling, less competition, and less quarreling than siblings of typical children. Interestingly, siblings of autistic children reported less prosocial behavior than siblings of children with DS, but *not* compared to siblings of typical children.

Walton and Ingersoll (2015) investigated sibling relationships in families with and without autism (n = 69; n = 93 accordingly), relying on maternal reports and using the Sibling Inventory of Behavior (SIB; Schaefer & Edgerton, 1981), which refers to rivalry, aggression, avoidance (grouped to represent negative behaviors), and empathy, teaching/directiveness, and companionship/involvement (grouped to represent positive behaviors) in the sibling relationships. They found no between-group differences in the overall maternal reported positive or negative behaviors in the sibling relationships. However, mothers reported siblings of autistic children to display lower levels of involvement, less aggression toward their siblings, and higher levels of avoidance of their siblings than mothers in the typical group. The authors concluded that sibling relationships in families of autistic children are not ‘better’ or ‘worse’ but might be characterized by different patterns than those of typical siblingships (Walton & Ingersoll, 2015). Qualitative research provides additional evidence on positive experiences alongside difficulties of siblings of autistic individuals and describes siblingship characteristics that are not always captured using standardized questionnaires (e.g., Mascha & Boucher, 2006; Petalas et al., 2012). For example, Benderix and Sivberg (2007) reported a sense of precocious responsibility and empathy among siblings of autistic children.

### Mothers’ and Siblings’ Perspectives

The studies reviewed above used not only different tools (SRQ, SIB), but also different informants (siblings, mothers). Still, there were some similarities in their findings: Kaminsky and Dewey (2001) found that siblings of autistic children and siblings of typical children did not differ in reporting overall closeness, power, and rivalry with their siblings, and Walton and Ingersoll (2015) found no difference in maternal reports of positive or negative behaviors in siblingships. Siblings of autistic children in Kaminsky and Dewey’s study reported less intimacy and nurturance with their siblings than siblings of typical children, which resembles Walton and Ingersoll’s findings of lower levels of involvement and more avoidance in the sibling relationships in the autism group, according to mothers. Importantly, similarities do not necessarily reflect identical perspectives. Different perspectives might also result in divergent results. For example, Rivers and Stoneman (2003) studied siblingships in 50 families of autistic children, using the SIB and a questionnaire measuring satisfaction with the relationships. They collected reports from mothers and typical siblings (ages 7–12) and found that the children were quite positive in the ratings of their relationship with their autistic siblings and expressed satisfaction with their siblingships, while parents' reports about the siblingship were less positive.

### The Present Study

Taken together, this literature suggests that available studies either: (1) focused on “positive” vs. “negative” or “better” vs. “worse” siblingships and not on specific qualities in the siblingships; or (2) referred to only parental or only siblings’ perspectives; or (3) did not include a comparison group of typical siblingships. The present study sought to add to the literature by addressing these points and investigating similarities and differences in sibling relationships in families of autistic and typical children, according to the typical siblings' and the mothers' perspectives, using a mixed methods approach. We aimed to explore whether warmth and closeness, conflict, rivalry, and power differ in siblingships with or without autism, (1) according to the typical sibling reports? (2) According to the mothers' reports? And (3) do siblings’ and mothers’ perspectives on the siblings' relationships converge or diverge? Lastly, we wanted to qualitatively investigate the key themes emerging from the participants' reports of the sibling relationship.

## Methods

Ethical approval was granted by Tel Aviv University. Data were collected as part of a larger project examining siblingships and autism (see also: Rum et al., 2021). Quantitative data were collected using self-report questionnaires, and qualitative data were collected through open-ended interviews using a mixed-methods convergent design (Creswell & Plano Clark, 2018). The quantitative and qualitative strands were implemented in a parallel approach, with equal emphasis (Bergin, 2018). Analyses were conducted separately, with an overall intention to draw conclusions based on the results from the two datasets.

### Participants

Seventy-five Israeli Hebrew-Speaking families participated. Twenty-nine typical children whose younger siblings had a diagnosis of autism and their mothers constituted the ‘autism group.’ Forty-six typical children with younger typical siblings and their mothers constituted the ‘typical group’. There were no significant differences between the two groups' demographic characteristics (see Table 1, results, and Supplementary). In the autism group, all younger siblings had a diagnosis of autism from authorized medical/health-care centers, where the ADOS (Autism Diagnostic Observation Scales; Lord et al., 1999; ADOS 2-Lord et al., 2012) is a part of the diagnostic battery. The autistic children were schooled in special education or inclusion programs in mainstream educational settings with an aide; Vineland Adaptive Behavior Scales (VABS; Sparrow et al., 1984, 2005) scores of the autistic children were varied, with a mean score of 81.07 (*SD* = 15.547; range 56–127). The sample included two pairs of twins in each group. The typical siblings in the autism group and participants and their siblings in the typical group were reported by their parents as having no health or developmental conditions and attending regular education schooling.

**Table 1:** Characteristics of study samples

	Typical Group *n* = 46 families	Autism Group *n* = 29 families
Participating Mothers (n = 75)		
Age in years, *M* (*SD*)	37.33 (3.83)	39.1 (5.32)
Age range	30–43	30–55
Maternal education, *n* (%)		
High-school	46 (100%)	29 (100%)
Higher education	46 (100%) – Bachelors Degree 15 (32.6%) – MA 21 (45.7%) – Ph.D. 4 (8.7%) – Higher edu. professional qualification 6 (13%)	27 (93%) – Bachelors Degree 11 (37.9%) – MA 9 (31%) – Higher educational professional qualification 7 (24.1%)
Family income level (*SD*)	4.04 (.83)	3.61 (1.16)
Number of household members		
4 or less than 4	7 (15.2%)	9 (31%)
5 or more than 5	39 (84.8%)	20 (69%)
Participating Children (Older typical sibling; n = 75)		
Sex, *n* (%)		
Male	17 (37%)	14 (48.3%)
Female	29 (63%)	15 (51.7%)
Sex in relation to the younger sibling *n* (%)		
Same-sex	20 (43.5%)	12 (41.4%)
Different sex	26 (56.5%)	17 (58.6%)
Age in years, *M* (*SD*)	8.78 (2.05)	9.12 (2.06)
Age range	6–12.02	4.42–12.39
Position in the family		
Firstborn	38 (82.6%)	22 (75.9%)
Not firstborn	8 (17.4%)	7 (24.1%)
Younger siblings		
Sex, *n* (%)		
Male	23 (50%)	25 (86.2%)
Female	23 (50%)	4 (13.8%)
Age in years, *M* (*SD*)	6.49 (1.58)	6.37 (1.52)
Age range	4–10.13	4.42–9.11
Position in the family		
Youngest in their families	12 (26.1%)	11 (37.9%)0
Not the youngest	34 (73.9%)	18 (62.1%)
Siblings' age gap in years, *M* (*SD*)	2.46 (0.92)	2.72 (1.36)

Mothers were requested to describe their household income by choosing one of five categories between 1 (a range of monthly amount reflective of an income level that is a lot below the national average) to 5 (a lot above the national average). The most common choice in both groups was reflective of an average (or slightly under/above the average) income for households in Israel

Recruitment was conducted through telephones to families enrolled in research databases at a center that provides autism diagnosis and treatment services and through adverts placed on social media, parent support organizations, and word of mouth. Inclusion criteria for the autism group were families with at least two children, in which the younger sibling has a diagnosis of autism, and the older sibling is a typical child. If there were more than two children in the family, the older sibling asked to participate was the sibling closer in age to the autistic child. Similarly, in the typical group, the older siblings participating were closest in age to the siblings they referred to in their reports in case of more than two siblings in the family.

### Data Collection

Parents signed an informed consent form and completed a demographic questionnaire. Older siblings and mothers in both groups completed a questionnaire and participated in an interview. Children were asked to read the first example question aloud to ensure reading ability, and the first two example items were completed in the researcher’s presence. For children that were judged to be pre-literate, a graduate student read aloud each question and the answer options.

### Materials

#### Sibling Relationship Questionnaire (SRQ; Furman & Buhrmester, 1985)

The brief version of the SRQ is a 39-item questionnaire in which responders are asked to rate how well a characteristic described the sibling relationship on a 5-point Likert scale (1- “hardly at all” to 5- “extremely much”). Both child’s self-report and parental-report versions are available; For example, an item from the parental-report version:

Some siblings do nice things for each other a lot, while other siblings do nice things for each other a little. How much do both _______ and *this sibling* do nice things for each other?

The parallel item in the child self-report version:

Some siblings do nice things for each other a lot, while other siblings do nice things for each other a little. How much do you and *this sibling* do nice things for each other?

The items cluster into four factors: (1) Warmth & Closeness (includes the subscales: intimacy, prosocial behavior, companionship, similarity, admiration by sibling, admiration of sibling, and affection); (2) Conflict (quarreling, antagonism, competition); (3) Rivalry (the average of maternal and paternal partiality); and (4) Relative Status Power (nurturance of sibling and dominance of sibling, minus the scale scores of nurturance by sibling and dominance by sibling). Buhrmester and Furman (1990) reported Cronbach's α for the SRQ that ranges from .71 to .81. For the current sample, Cronbach's α ranged from 0.7 to 0.9, with two exceptions: maternal reported maternal partiality (α = .54) and siblings’ reported dominance by sibling (α = .6).

#### Open-Ended Interview

Most participants (84%) also agreed to participate in a short open-ended interview. Children (n = 65) were asked: “Tell me about yourself, about < *the sibling* > and about your relationship”, mothers (n = 61) were asked: “Tell me about < *the younger sibling* > and his sibling, and about their relationship.” Mothers were offered to either speak to a recording device or write their answers. All children were recorded, giving their answers orally. All responders were asked to speak (or write) for up to 5 min uninterrupted, with no other guiding question on the part of the interviewer that was present to record the answers (similar to the Five-Minute Speech Sample method; FMSS; Taylor & DaWalt, 2017; Woodman et al., 2015). When they had completed their answers and posed, the interviewer asked if they had anything to add, and the interview stopped when the participants stated that they had nothing else to say. No participant reached the 5 min time limit. The longest recorded answer was 4:51 min long, and the shortest was 1 min long of free, uninterrupted speech. Each recorded interview was transcribed, and texts were qualitatively analyzed.

### Data Analysis

Statistical analyses were conducted using IBM SPSS 25 and RStudio based on R software. Content analyses were conducted using Microsoft Excel. Descriptive statistics were computed for demographic variables (Table 1), and analyses of variance (ANOVA) and χ^2^ tests were conducted to examine whether there are between-group differences in demographic characteristics.

#### Questionnaires

Multivariate analyses of variance (MANOVA) were used to determine the presence of between-group differences in the SRQ scores, according to the two informants. T-tests were used to investigate the direction of parental preference in the items that compose the rivalry factor, as these items are structured as a comparison between the older and the younger sibling, with the middle choice indicating neutrality. Any answer different than 3 in these items indicates a preference for a particular child; therefore, we compared the answers to the middle value indicating no partiality (3).

Pearson correlation tests were used to measure correlations between mothers' and children's reports (SRQ scores) within each group and potential correlations between the siblings’ age and the SRQ measures. We used a p < 0.05 cutoff for all values for significance.

#### Open-Ended Interview

An inductive, data-driven approach was used to analyze the interviewee's answers. A qualitative analysis was used to identify patterns of information within the data, which converged into key elements of the participants' perspectives of the sibling relationship (Birks & Mills, 2015). Texts were divided into basic units for analysis—quotes. A quote was defined as a statement that expressed one central idea and was separated from quotes before or after hand by either a significant break in the flow of speech, by changing the main idea expressed, or both (Ayalon & Sabar-ben Yehoshua, 2010). Prior to analysis, each participant's text was assigned a number (participant ID), and each quote was assigned a serial number from 1 to n. Thus, every quote had a number indicating its source.

An Excel sheet was used to group quotes with similar sentiments into categories. The analysis began with open coding, where primary repeated patterns in the data were located. Data extracts and initial categories suggested by the first author (YR) were audited by the last author (ED), and disagreements were discussed. When categories were finalized, axial coding and entry criteria for each category were formulated, and then a directed coding analysis continued until all the data was classified into categories. A theoretical integration was then made, and the finalized set of categories was grouped into sub-themes and then into themes, with both authors refining themes until a consensus was reached (Birks & Mills, 2015; Sabar-ben Yehoshua, 2016). Two mothers and one sibling from the autism group reviewed a summary of the main finding and conclusions provided by the first author and provided feedback that was considered in composing the manuscript.

## Results

No significant between-groups differences were found in the demographic characteristics maternal education (*F*_(1,73)_ = 2.78, *p* = 0.10), mothers’ age (*F*_(1,73)_ = 2.82, *p* = 0.09), participating children’s age (*F*_(1,73)_ = 0.17, *p* = 0.68), younger siblings’ age (*F*_(1,73)_ = 0.05, *p* = 0.82), siblings’ age gap (*F*_(1,73)_ = 0.98, *p* = 0.33), reported family income level (*F*_(1,69)_ = 3.09, *p* = 0.08), number of household members (*F*_(1,73)_ = 0.75, *p* = 0.39), and older siblings' sex (*χ*^2^_(1, N=75)_ = 0.94, *p* = 0.35). As expected, a significant difference was found in the younger siblings' sex (χ^2^_(1, *N*=75)_ = 10.12, *p* = 0.001), with more males than females among the younger siblings in the autism group and an equal number of males and females among the younger siblings in the typical group. The male:female ratio among the autistic siblings was similar to the ratio reported in Israel (Raz et al., 2015). Due to the relatively young age of some of the participants, we also examined associations between the age of the siblings and the sibling relationship scales reported by mothers and siblings (see Supplementary). No significant correlations were found, apart from a moderate negative correlation (*r*_(44)_ =  − .31, *p* = .037) between the older typical sibling’s age and the rivalry score reported by siblings in the typical (but not the autism) group. Older age of the reporting sibling in the typical group was associated with a lower level of siblings’ rivalry. As another measure of caution, we also repeated the reported analyses of between-group comparisons, excluding the youngest participant in the sample (one under 6 years old sibling in the autism group), and the results remained similar to those reported in the next section, based on the whole sample.

### Questionnaires

#### Between-Group Differences

Table 2 presents descriptive statistics. According to the children's reports, no between-group differences were found in the SRQ factors: warmth/closeness (*F*_(1,72)_ = 1.403, *p* = .24, *η*^2^ = .019); conflict (*F*_(1,72)_ = 2.87, *p* = .095, *η*^2^ = .038); rivalry (*F*_(1,72)_ = 2.133, *p* = .149, *η*^2^ = 0.029); relative status power (*F*_(1,72)_ = 0.22, *p* = .642, *η*^2^ = 0.003). Differences were found in two of the SRQ subscales: Siblings of autistic children reported less intimacy (*F*_(1,72)_ = 6.08, *p* = .016, *η*^2^ = .078) and quarreling (*F*_(1,72)_ = 4.27, *p* = .042, *η*^2^ = .056) than siblings in the typical group.

**Table 2:** Means and Standard Deviations of Mothers and siblings reports on the four SRQ factors (bold), and on each subscale

	Siblings' Reports					Mothers' Reports			
	Typical Group		Autism Group			Typical Group		Autism Group	
	M	SD	M	SD		M	SD	M	SD
Warmth and Closeness	3.29	0.93	3.10	0.74	Warmth and Closeness*	3.42	.65	3.02	.79
Intimacy*	2.79	1.42	2.07	1.09	Intimacy**	2.78	1.05	1.79	.95
Prosocial	3.34	1.01	3.14	0.95	Prosocial*	3.43	.79	3.05	.79
Companionship	3.7	1.05	3.41	0.92	Companionship	3.82	.93	3.5	1.02
Similarity	2.98	1.09	2.61	1.05	Similarity**	2.46	.75	2.71	.95
Admiration of Sibling	3.14	1.17	3.33	1.16	Admiration of Sibling	3.82	.91	2.81	1.21
Admiration by Sibling	3.37	1.13	3.04	1.36	Admiration by Sibling	3.46	.74	3.31	1.47
Affection	3.66	1.08	3.91	0.98	Affection	2.78	.84	3.97	.9
Conflict	2.81	0.86	2.52	1.10	Conflict*	3.03	.8	2.56	1.03
Quarreling*	3.24	0.91	2.9	1.31	Quarreling*	3.44	.88	3.02	1.19
Antagonism	2.64	1.00	2.35	1.22	Antagonism	2.68	.98	2.31	1.17
Competition	2.59	1.23	2.28	1.33	Competition**	3.02	1.03	2.36	1.17
Rivalry	3.03	0.53	2.92	0.48	Rivalry	2.9	.27	2.79	1.03
Maternal partiality	3.03	0.65	2.77	0.62	Maternal partiality	2.81	.36	2.73	.48
Paternal partiality	3.04	0.55	3.00	0.66	Paternal partiality	2.99	.4	2.89	.49
Relative Status Power	2.09	1.525	2.16	1.10	Relative Status Power	1.99	1.6	2.46	2.03
Nurturance of sibling	3.31	1.03	3.37	0.94	Nurturance of sibling	3.46	.74	3.3	.97
Nurturance by sibling	1.88	0.94	1.93	1.08	Nurturance by sibling**	2.25	.7	1.79	.98
Dominance of sibling	2.41	0.93	2.57	1.02	Dominance of sibling	3	.85	3	.97
Dominance by sibling	1.76	0.69	1.81	0.84	Dominance by sibling	2.22	.91	2.05	.94

Mean differences in the SRQ scores were analyzed using multivariate analyses of variance (MANOVA). T-tests were used to investigate the direction of parental preference in the items that compose the rivalry factor, as these items are structured as a comparison between the older and the younger sibling, with the middle choice indicating neutrality. Any answer different than 3 in these items indicates a preference for a particular child; therefore, we compared the answers to the middle value indicating no partiality (3)

*p < .05; **p < .01

Mothers of autistic children reported their children to have less warmth/closeness (*F*_(1,72)_ = 5.63, *p* = .02, *η*^2^ = .073) and less conflict (*F*_(1,72)_ = 6.66, *p* = .012, *η*^2^ = .085), but similar levels of rivalry (*F*_(1,71)_ = 2.44, *p* = .123, *η*^2^ = 0.033) and relative status power (*F*_(1,72)_ = 2, *p* = .161, *η*^2^ = 0.027) compared with mothers in the typical group. Mothers in the autism group also reported less intimacy (*F*_(1,72)_ = 20.23, *p* = .00, *η*^2^ = .219), less quarreling (*F*_(1,72)_ = 4.94, *p* = .029, *η*^2^ = .064), less prosociality (*F*_(1,72)_ = 4.57, *p* = .036, *η*^2^ = .06), less sibling nurturance (*F*_(1,72)_ = 8.29, *p* = .005, *η*^2^ = .103), less similarity between the siblings (*F*_(1,72)_ = 14.2, *p* = .00, *η*^2^ = .165), and less competition (*F*_(1,72)_ = 7.42, *p* = .008, *η*^2^ = .093) in the siblings' relationships than mothers in the typical group.

##### Parental Partiality

According to the mothers' reports, in both groups, maternal partiality was significantly different from the neutral choice, demonstrating a preference for the younger sibling (*t*_(27)_ =  − 3.17, p = 0.004; *t*_(45)_ =  − 3.59, p = .001), while paternal partiality did not differ from the neutral choice. Children in the autism group also reported a maternal preference for the younger sibling (*t*_(28)_ =  − 3.15, p = .004) and no paternal partiality. In the typical group, children’s reports indicated no maternal or paternal partiality.

### Agreement Between Informants

Tables 3 and 4 show correlations between children's and mothers' reports. In the autism group, children's and mothers' reports correlated only in the conflict factor (*r*_(29)_ = .454, *p* = .0103). In the typical group, children's and mothers' reports correlated for conflict (*r*_(46)_ = .410, *p* = .004) and warmth/closeness (*r*_(46)_ = .472, *p* = .001). Other significant correlations were found ‘off the diagonal line,’ including a positive correlation between maternal reported conflict and siblings’ reported rivalry in the autism group and a negative correlation between maternal reported conflict and siblings’ reported warmth and closeness in the typical group.

**Table 3:** Correlations between informants in SRQ factors in the Autism group

Mothers	Siblings			
	Warmth and Closeness	Conflict	Rivalry	Relative Status Power
Warmth and Closeness	− .038	.293	.154	− .201
Conflict	− .009	.454*	.457*	− .348
Rivalry	− .068	.331	.288	− .393*
Relative Status Power	.034	− .230	− .286	.073

*p < .05

**Table 4:** Correlations between informants in SRQ factors in the typical group

Mothers	Siblings			
	Warmth and Closeness	Conflict	Rivalry	Relative Status Power
Warmth and Closeness	.472**	− .281	.203	.012
Conflict	− .297*	.410**	.173	.011
Rivalry	− .010	.144	.136	− .225
Relative Status Power	.035	− .269	.070	.208

*p < .05; **p < .01

### Open-Ended Interview

Table 5 presents the subthemes identified in the qualitative analysis, grouped into four main themes: Table 6 summarizes the main themes by informants and groups. Two themes were common to mothers and children in both groups: (a) ‘Inseparable’, (b) ‘The younger versus older’; one theme was unique to mothers in both groups: (c) ‘The bigger picture’; and one theme was unique to mothers and siblings only in the autism group: (d) ‘The unsaid word’.

**Table 5:** Sub-themes by informants and groups

Theme	Sub-Theme	Mothers		Siblings	
		Typical*** n*** = 38 Participants (195 quotes)	**Autism *n*** = 23 (140)	Typical*** n*** = 38 (132)	**Autism *n*** = 27 (156)
Inseparable	Equality in the relationship	**9** (10)			
	Joint routine and things in common	**19** (24)	**10** (13)	**16** (19)	**6** (9)
	Good relationship, joy	**18** (30)	**14** (18)	**22** (39)	**11** (21)
	Love and affection	**9** (10)	**7** (10)	**5** (6)	**7** (14)
	Quarrels and fights	**8** (9)	**8** (8)	**10** (10)	**10** (15)
	“The good and the bad come together”	**17** (18)	**4** (4)	**19** (26)	**8** (13)
The younger vs. the older	Rivalry, competitiveness and jealousy	**10** (13)			
	Bad relationship, distance	**2** (3)	**3** (5)	**6** (14)	**2** (3)
	Discrepancies, differences and a-symmetry	**13** (19)	**10** (11)	**2** (4)	**6** (7)
	Parental attention		**4** (4)	**1** (2)	**3** (4)
	The older sibling role	**8** (9)	**9** (12)	**2** (2)	**11** (14)
	The older sibling’s power (and younger sibling’s admiration of the older sib)	**7** (7)	**6** (7)	**1** (1)	**5** (6)
	Difficulties and needs of the older sibling/ “My sibling hurts/disturbs me”	**3** (3)		**5** (8)	**7** (12)
	Strengths of the younger sibling's	**4** (5)	**4** (4)	**1** (1)	**5** (5)
	Strengths of the older sibling	**4** (4)	**4** (5)		
	Difficulties due to the younger sibling's condition	**3** (5)	**4** (7)		
The bigger pic	Changes and processes in the relationships	15 (17)	11 (13)		
	The parents' role and the parents’ efforts		**4** (6)		
	Parental/adult intervention interrupts the relationships	**8** (9)			
The unsaid word	*Explicit terminology (“on the spectrum”/”autistic”)*		**1** (1)		**1** (1)
	The younger sib. embarrasses the older sib		**4** (4)		
	Special abilities of the older sibling to understand and motivate the younger sib		**7** (8)		
	“My sibling is different.”				**8** (10)
	“My sibling is not different.”				**3** (5)
	“My sibling behaves in a way I cannot understand/explain.”				**9** (9)
	“My sibling seeks to be close to others.”				**6** (8)

*n*’s, in bold, represent the **number of participants contributing quotes to the subtheme** (the total number of quotes in the subtheme in brackets)

**Table 6:** Themes by informant and group

Group	Informant	
	Mothers	Children
Typical	(a) ‘Inseparable’ (b) ‘The younger versus older.’ (c) ‘The bigger picture.’	(a) ‘Inseparable’ (b) ‘The younger versus older.’
Autism	(a) ‘Inseparable’ (b) ‘The younger versus older.’ (c) ‘The bigger picture.’ (d) ‘The unsaid word.’	(a) ‘Inseparable’ (b) ‘The younger versus older.’ (d) ‘The unsaid word.’

Theme (a) ‘Inseparable’, included references to the siblings' joint routine, things in common, joy, affection, and conflicts, and statements on how these ‘come together’ (e.g., *“we fight a lot and we have much fun together” [participant’s age*: 10 years old*]; “they love, they fight, they play, as all siblings do”*). At the early stages of coding, categories relating to joint routine and things in common, good relationships and joy, love, affection, and quarrels and fights clearly emerged from the data. However, many quotes remained unclassified since they seemed to suit both a category describing the closeness and warmth in the sibling relationship and the conflicts between the siblings at the same time. For example, quotes consist of expressions of affection or joy alongside expressions of dislike or anger or descriptions of fights and quarrels that cannot be separated into two different sentences. In addition, a recurring pattern in the data was that, in many cases, the same interviewee provided separate quotes for one or more of the categories related to warmth and closeness in the siblingship and the category of conflicts in the same short answer. One interviewee, 9 years old, said that “*that's how siblings are*” and that “*no one can separate us*,” and another (10 years old) noted that “*the good and the bad come together*” in a sibling relationship, quotes that led to the decision to theoretically group these sub-themes into a theme relating to how these elements do come together in sibling relationships.

Theme (b) ‘The younger versus older’, included references to each sibling's role, disparities, and asymmetry in the relationship (e.g., “*they have very different characters*”; “*I help him with things that he doesn't know*”* [participant’s age: 6.5 years old]*). Within this theme, one sub-theme was unique to mothers in the typical group and included references to rivalry, competitiveness, and jealousy between the siblings. Interestingly, another sub-theme unique to mothers in the typical group included references to equality in the sibling relationship. In other words, no interviewees in the autism group (mothers or siblings) and no siblings in the typical group referred to these aspects of sibling relationships in their answers to the open question.

Theme (c) ‘The bigger picture,’ included subthemes references to changes and processes in the sibling relationship that also involve other events or people and their impact on the siblingship (e.g., *“I assume that the improvement in their relationship is a result of [the autistic child]’s progress in language*”; “*their relationship is worse since their sister was born*”). Interestingly, this theme included two contradictory sub-themes regarding the parent’s role: A sub-theme that emerged in the typical group included references to less parental involvement as good for the siblingships (e.g., “*when we are not around, they play and collaborate wonderfully*”; “*they fight and tease each other, mostly when mommy or daddy are around*”); a sub-theme in the autism group included quotes from mothers saying that good siblingships result from “*lots of work, explanations, and support by us, the parents*.”

Specific to the autism group, theme (d) was titled: The unsaid word’. It grouped sub-themes referring to the younger sibling's condition: children describing their siblings as ‘different’ (“*he is not like any ordinary brother*” *[participant’s age: 10.5 years old]*) or ‘not different’ (“*we are a special family, with a special child, and another special child [refers to herself; participant’s age: 9 years old]*”), and references to behaviors that they ‘cannot explain’ (“*sometimes I find his behavior weird… I have no idea what he is doing and why*” *[participant’s age: 10.5 years old]*), mothers’ descriptions of their typical children’s embarrassment *(e.g., “he sometimes feels embarrassed by her behavior in public places or in front of people we know”*), alongside their children’s abilities to understand and motivate their autistic siblings (*“she can understand him without words”; “she doesn’t feel sorry for him or gives up, and at the same time is aware of his special needs*. Interestingly, these references did not include the term ‘autism’ (or ‘autistic’/ ‘spectrum’), except for by one sibling (“*I don’t know if my mother told you, but he is autistic.*” *[participant’s age: 12 years old])*, and his mother (“*the ‘little one’ is ‘on the spectrum*”).

For an overview of the mixed-methods results, see Table 7, which summarizes themes and quantitative differences in the sibling relationship.

**Table 7:** Mixed-methods results

Qualitative finding (Themes)	Quantitative finding (SRQ)
(a) ‘Inseparable’ *A theme found in both groups, both informants*	Mothers of autistic children reported their children to have less warmth/closeness and less conflict in the typical group No between-group differences were found in the SRQ factors: warmth/closeness, and conflict Mothers: Autism < Typical; Siblings: No difference
(b) ‘The younger versus older.’ *A theme found in both groups, both informants*	No between-group differences were found in the SRQ factors: rivalry, relative status power Mothers and Siblings: No difference
(c) ‘The bigger picture.’ *A theme found in both groups*	More between-group differences were found in SRQ mothers’ reports than in siblings’ reports
(d) ‘The unsaid word.’ *A theme found in the autism (and not the typical) group, both informants*	More disagreement between mothers’ and siblings’ reports in the autism group than in the typical group Common to mothers and siblings: Less reported intimacy in the sibling relationship and less reported quarreling (SRQ subscales) in the autism group than in the typical group

## Discussion

The mixed methods multi-informant examination of what is similar and different in the relationships of autistic children and their siblings compared with typical siblingships revealed not only differences and similarities in the sibling relationships but also in mothers' and children's perspectives. Overall, siblings of autistic children reported a typical-like siblingship, with high levels of conflict alongside warmth and closeness in the siblingship, while mothers' reports portrayed a more distant or uninvolved siblingship. When describing the siblingship in their own words, siblings (the children) were more focused on the “here and now” siblingship experiences, and mothers referred more to processes in the relationships that also included other people and events. In the following section, we discuss the findings and their implications.

The present findings indicate mainly similarities between siblings of autistic and typically developing children in reporting on their sibling relationships, with no between-group differences in overall warmth, conflict, rivalry, or power. More differences were found in mothers' reports on siblingships: mothers of autistic children reported less warmth and closeness and less conflict between their children than mothers of only typical children. The qualitative findings suggest that while children focused on “here and now” experiences, mothers also referred to changes and processes. This difference in perspectives is aligned with an expected developmental difference between children who might be more focused on the present, whereas adults potentially have a firmer grasp on change over time. This difference is important to note, considering the fact that in the sibling relationship literature, some findings are based on children’s reports, and others are based on parents’ reports (see introduction). It might also account for the fact that more between-group differences were indicated according to mothers’ reports than according to siblings’ reports in the current study. It could be that while the day-to-day experience of being a sibling to an autistic child is somewhat similar to a typical siblingship experience, a wider perspective on the relationships reveals more differences in the sibling relationships (or at least, mothers’ perspectives on these relationships) between families with and without autism. In other words, the differences between children and mothers in their focus might account for the fact that there were more between-group differences in the reports of mothers (mothers of only typical children in comparison to mothers of typical and autistic children) compared to between-group differences in the reports of children (siblings of typical children in comparison to siblings of autistic children). In addition, it could be that while children grow up into a siblingship natural to them, parents are more influenced by expectations and comparisons to other relationships. Another explanation for the different views on the sibling relationship between siblings and mothers might be that mothers project their feelings when reporting on their children's relationships. For example, it was found that mothers' evaluations of their autistic children's anxiety were affected by their own anxiety (Bitsika et al., 2015, 2021).

Despite the differences in mothers’ and children’s perspectives, reports converged on some similarities and differences between siblingships with and without autism. Mothers’ and children's reports indicated similar overall rivalry and relative power, ranking the older siblings as more dominant in the relationships. However, the present study included only sibling dyads in which the younger sibling is autistic, and it might be that this commonality would not be followed for sibling pairs in which the autistic child is the older sibling.

In the qualitative strand of the current study, many interviewees referred to warmth and closeness and conflicts between siblings, two fundamental characteristics of the sibling relationship in middle childhood (Buhrmester & Furman, 1990; Buist & Vermande, 2014). Some participants explicitly mentioned that these qualities, the “good and the bad,” come together. Such references were recorded from mothers and siblings, from families with and without autism, indicating an important similarity between sibships with and without autism. Expressions of both positive and negative emotions and feelings in both groups correspond with Campione-Barr and Killoren’s theoretical claim that the coexistence of positive and negative feelings toward one another is a unique hallmark of siblinghood. While such ambivalence in other close relationships (e.g., parent–child relationships, friendships, romantic relationships) would be suboptimal, the ambivalent nature of siblingship is both relationally and developmentally appropriate (Campione-Barr & Killoren, 2019). Importantly, the qualitative findings of the present study suggest that this ambivalence characterizes relationships in which one of the siblings has autism, as it characterizes typical siblingships. Common to both informants and groups and resonant with the quantitative finding of the dominance of the older sibling were also references to discrepancies, differences, and a-symmetry in the siblings' relationship and each sibling’s role in the qualitative data.

The quantitative findings indicated two differences reported by mothers and siblings: less intimacy and less quarreling between the siblings' pairs in which one of the siblings is autistic, compared with typical siblingships. Mothers of autistic children reported lower levels of overall warmth and of conflict in their children's relationships, a pattern that was previously termed as distant or uninvolved siblingship type (Derkman et al., 2011; Sherman et al., 2006; Whiteman & Loken, 2006) and was found to be less common in middle childhood (Buist & Vermande, 2014). The children’s reports converged with those of the mothers only for less quarreling and intimacy and did not indicate an overall uninvolved pattern. However, it could be that the less quarreling in siblingships with autism, as indicated by both mothers' and siblings’ reports, is a result of less intimacy and sibship involvement. In other words, it could be that these siblings have fewer quarrels than typical siblings because they interact less. In future studies, it will be interesting to measure the length, intensity, and quality of siblings’ interactions in families with and without autism.

The qualitative analysis also offers possible explanations for these quantitative findings of less quarreling in sibships in the autism group. The sub-themes relating to rivalry, competitiveness, and jealousy between the siblings, and equality in the sibling relationship were identified only in the reports of mothers of typical children. The absence of these sub-themes from the texts of mothers of autistic children suggests that these qualities might be less prominent in siblingships in which one child is autistic, as siblings are (or mothers perceive them to be) less similar to each other. Therefore, older, typical siblings might feel quarreling would be ineffective in an imbalance conflict. Some mothers described their typical children as having special abilities to understand their younger autistic siblings. Perhaps the older siblings avoid quarreling to benefit their younger siblings or to protect them. It could also be that carers encourage children to avoid quarrels with their autistic siblings. On the other hand, it could also be that conflicts between the siblings are less verbal or involve communication difficulties. The qualitative results indicate that some children find it hard to understand their autistic siblings’ behaviors. If the siblings have a conflict but struggle to communicate, this might result in negative emotions that do not necessarily involve quarrels.

Communication difficulties might also account for reduced intimacy in the siblingship. As the items measuring intimacy in the questionnaire (SRQ; Furman & Buhrmester, 1985) focus on sharing emotions and secrets, reduced intimacy might result from difficulties of the autistic children in Theory of Mind abilities or difficulties in self-recognition of emotions that are reported among autistic individuals (Ben-Itzchak et al., 2018). It could also result from the difficulty of the typical siblings in understanding the way their autistic siblings might experience and express emotions in a different (not typical) way (Jaswal & Akhtar, 2019). It would be interesting to further investigate what autistic and non-autistic siblings feel about intimacy in their relationship and examine factors that might enhance intimacy in the siblingship. For example, disclosure to siblings was found to be associated with positive feelings, greater trust, and emotional support in typical sibling relationships (Howe et al., 2000, 2001). It could be that encouraging siblings to disclose their feelings to each other or even talk about the siblings’ autism will benefit the relationships.

The fact that during the interview, only one child and one mother used explicit terminology (‘autism’/’spectrum’) to refer to the younger sibling’s diagnosis was striking. Siblings of autistic children are likely to learn about autism through adults in their lives, particularly parents (Macedo Costa & Pereira, 2019), and this might imply the degree of comfort or the frequency of using or not using explicit terminology in the family. There could be different reasons for that, like shame or embarrassment, or, on the other hand, acceptance of autism as a plain fact that there was no need to mention. Improved knowledge of autism was found to be associated with a better sibling relationship (Roeyers & Myche, 1995; Jones et al., 2019). Some children mentioned in the interviews that they have difficulties understanding their sibling’s behavior. A better understanding of autism characteristics might reduce such frustrations. Interestingly, Coffman et al. (2021) found that knowledge of autism was also associated with more sibling reports of aggressive behaviors of their autistic siblings and speculated it might be explained by more awareness or a greater need for explanations of autism in cases where the autistic child exhibits aggressive behaviors. They also suggested that perhaps simple knowledge of the characteristics of autism may be insufficient to promote satisfaction with the siblingship, and a more nuanced understanding is needed, including how siblings might show their care and affection in other ways (Jaswal & Akhtar, 2019). Disclosure of autism in the family and the siblings’ knowledge of autism might play a role in the results of our study and should be addressed in future replications.

The findings also indicate that mothers of typical siblings report more sibling conflict when the typical siblings themselves report lower levels of warmth and closeness in their relationships. However, this association was not observed in the autism group. Instead, in families with autism, mothers' reports of higher conflict between siblings were linked to higher levels of rivalry reported by the siblings. These results suggest potential differences in how sibling conflicts manifest in families with and without autism, or in how mothers interpret behavioral information to assess the level of conflict between their children.

The findings of this study should be evaluated in light of its limitations. First, the conclusions cannot be generalized to sibling pairs in which the autistic child is the older sibling. While we focused on sibling pairs of older typical siblings and younger autistic siblings, we encourage future research focusing on siblingships in which the older sibling is autistic, and also on siblingships in multiplex families with more than one autistic child. In addition, autism is a varied spectrum, and directed research of sibship in dyads with specific characteristics of the autistic siblings is needed, for example, sibling dyads that include minimally verbal autistic children. Specific characteristics of autism could potentially impact sibling relationships in different ways. It is likely that the relationship dynamics would differ between, for instance, a minimally verbal autistic individual with high support needs and their sibling and the relationship of an intellectually gifted but rigid autistic individual and their sibling. A future study could delve deeper into these nuances, which will be helpful not only on the theoretical level but also in planning tailored support for individuals, sibling dyads, and families when needed.

It is important to focus in future studies on the perspectives of autistic siblings on the sibling relationship. Petalas et al. (2015) conducted a qualitative interpretative phenomenological analysis of semi-structured interviews with 12 autistic adolescents and found that the autistic adolescents reported seemingly typical sibling interactions and, at the same time, did refer to how these typical sibling interactions are influenced by having an autism spectrum condition. Future studies in quantitative or mixed methods approaches are clearly needed to shed more light on how autistic siblings perceive the sibling relationship and on similarities and differences in the perspectives of the autistic and the typical siblings, as well as parents and other family members. Moreover, considering the double empathy theory and accumulative research supporting it (Crompton et al., 2020a, 2020b; Morrison et al., 2019), exploring the nature of a siblingship of two autistic individuals will be of great value.

A second limitation of the present study is the use of a volunteer sample that might be skewed towards families with positive attitudes towards research. Since this study was part of a larger project that included home visits and observations on sibling interaction (see Rum et al., 2021), participation was time-consuming and posited openness and high involvement on the part of each family. It is important to take this context into account in interpreting the findings. This study design also did not allow examination of the developmental aspects of sibling relationships, as it did not include longitudinal data from more than one measurement point or large enough groups of participants in various age groups. The age of the siblings in our sample was not associated with the measures of qualities of the sibling relationships, with one exception: typical siblings in typical families reported less siblings’ rivalry as they were older in age. Interestingly, this correlation was not found for siblings of autistic children. Importantly, the investigation of changes in the siblings’ relationship and changes in perspectives on these relationships is an important direction for future research.

Another limitation relates to the small number of autistic female siblings in our sample, which made it impossible to draw conclusions on possible differences between same- versus opposite-sex sibling pairs. The present sample also did not allow the exploration of potential differences between the experience of having an autistic sister and having an autistic brother. Considering the accumulation of knowledge on the autistic female phenotype (Allely, 2019), we emphasize the importance of studying this topic of sibships that include an autistic sibling in future research. In addition, the older (typical) siblings were included based on parental reports, and we believe that future studies should directly assess typical siblings for cognitive and social measures that might influence the sibling interactions, such as, for example, the broader autism phenotype (BAP). In future studies, we also suggest addressing the quantitative assessment of maternal reported partiality and siblings’ reported dominance by siblings, which reached lower internal consistency values (Cronbach's α; .54, .6 respectively) than other SRQ scales in our sample.

Notwithstanding these limitations, this multimethod, multi-informant study carries theoretical and practical significance in providing an updated examination of siblingships in the context of autism. It highlights a generally positive experience for children with younger autistic siblings that mostly follow typical sibling relationship patterns according to their own reports. At the same time, mothers’ perspectives provide more context and emphasize differences and challenges in such siblingships, reporting a pattern of decreased involvement. It is important to consider both perspectives in research and in clinical work with families of autistic children.

This study also points to some important future directions in the research of sibling relationships and autism. The similarities and differences found in siblings' and mothers’ perspectives on the sibling relationship emphasize the importance of focusing on intimacy in the sibling relationship, and the possible different nature of siblings’ conflicts, as well as the need for direct observation of sibling interactions and longitudinal designs to investigate changes in the relationships and in the siblings’ perspectives over time. Studies to investigate the role of sex, emotional challenges to siblings as well as benefits from siblingships are also future directions that will allow a fuller and deeper understanding of this lifelong, meaningful relationship between autistic individuals and their siblings.

## Supplementary Information

Below is the link to the electronic supplementary material.

Supplementary file1 (DOCX 125 KB)
