# Supplementary material for: Sibling Relationships in Families of Autistic and Typical Children: Similarities and Differences in the Perspectives of Siblings and Mothers
Source: J Autism Dev Disord. 2024 Jan 20;55(2):620–34. doi: 10.1007/s10803-023-06222-0 (PMC11814030; doi:10.1007/s10803-023-06222-0)
Supplement: Supplementary file 1 — Supplementary file1 (DOCX 125 KB) [file 10803_2023_6222_MOESM1_ESM.docx]

**Supplementary**

Table 8. Correlations for siblings’ ages and SRQ factors by informants in the Autism group

|  |  | Sibling’s age  RQ factor | Older sibling’s age | Younger sibling’s age |
| --- | --- | --- | --- | --- |
|  | Mothers’ reports | Warmth & Closeness | *r*_(27)_ = -.16; *p* = .408 | *r*_(27)_ = -.09; *p* = .649 |
|  |  | Conflict | *r*_(27)_ = -.34; *p* = .074 | *r*_(27)_ = -.19; *p* = .331 |
|  |  | Rivalry | *r*_(27)_ = -.07; *p* = .727 | *r*_(27)_ = -.01; *p* = .974 |
|  |  | Relative Status Power | *r*_(27)_ = -.08; *p* = .679 | *r*_(27)_ = -.20; *p* = .295 |
|  | Older siblings’ reports | Warmth & Closeness | *r*_(27)_ = -.14 ; *p* = .459 | *r*_(27)_ = -.03 ; *p* = .892 |
|  |  | Conflict | *r*_(27)_ = -.34; *p* = .072 | *r*_(27)_ = -.22; *p* = .246 |
|  |  | Rivalry | *r*_(27)_ = -.16; *p* = 0.392 | *r*_(27)_ = -.23; *p* = 0.229 |
|  |  | Relative Status Power | *r*_(27)_ = -.06; *p* = 0.757 | *r*_(27)_ = -.20; *p* = 0.292 |

Table 9. Correlations for siblings’ ages and SRQ factors by informants in the typical group

|  |  | Sibling’s age  RQ factor | Older sibling’s age | Younger sibling’s age |
| --- | --- | --- | --- | --- |
|  | Mothers’ reports | Warmth & Closeness | *r*_(44)_ = -.27; *p* = .070 | *r*_(44)_ = -.10 ; *p* = .499 |
|  |  | Conflict | *r*_(44)_ = -.02 ; *p* = .888 | *r*_(44)_ = .02 ; *p* = .878 |
|  |  | Rivalry | *r*_(44)_ = .03 ; *p* = .826 | *r*_(44)_ = -.01; *p* = .974 |
|  |  | Relative Status Power | *r*_(44)_ = -.03 ; *p* = .679 | *r*_(44)_ = -.10; *p* = .500 |
|  | Older  siblings’ reports | Warmth & Closeness | *r*_(44)_ = -.13 ; *p* = .386 | *r*_(44)_ = -.14 ; *p* = .341 |
|  |  | Conflict | *r*_(44)_ = .01 ; *p* = .949 | *r*_(44)_ = .09 ; *p* = .550 |
|  |  | Rivalry | *r*_(44)_ = -.31 ; *p* = .036 | *r*_(44)_ = -.20 ; *p* = 0.178 |
|  |  | Relative Status Power | *r*_(44)_ = -.12 ; *p* = 0.414 | *r*_(44)_ = -.24 ; *p* = 0.112 |
